# Psychological distress among South African healthcare workers during the COVID-19 pandemic

**DOI:** 10.4102/curationis.v47i1.2477

**Published:** 2024-02-19

**Authors:** Shandir Ramlagan, Ronel Sewpaul, Yolande Shean, Tenielle Schmidt, Alicia North, Sasiragha P. Reddy

**Affiliations:** 1 Department of Human and Social Capabilities, Human Sciences Research Council, Pretoria, South Africa; 2 Registry of Senior Australians, South Australian Health and Medical Research Institute (SAHMRI), Adelaide, Australia; 3 School of Education, University of KwaZulu-Natal, Durban, South Africa

**Keywords:** psychological distress, healthcare workers, COVID-19, South Africa, mental health

## Abstract

**Background:**

The COVID-19 pandemic has placed immense pressure on healthcare workers (HCWs).

**Objectives:**

This study sought to find the prevalence and factors associated with psychological distress among HCWs in South Africa during the beginning phases of COVID-19 and make relevant recommendations.

**Method:**

The survey was administered online through a data-free platform. Data were benchmarked to the national population of over 500 000 healthcare professionals in South Africa. Multiple logistic regressions were used to determine association between psychological distress and potential explanatory variables.

**Results:**

A total of 7607 healthcare professionals participated in the study (1760 nurses, 2843 medical practitioners and 3004 other healthcare professionals). Half of the nurses, 41% of medical practitioners and 47% of other healthcare professionals were classified as psychologically distressed. Those who were of older age, provided with well-being support services and having a positive outlook on the healthcare system were significantly less likely to be distressed. Being female medical practitioners and female other healthcare professions, requesting routine counselling, being concerned about not having enough leave and that their life insurance policy did not cover COVID-19 were more likely to be distressed.

**Conclusion:**

Psychological well-being of HCWs in South Africa is at risk. We recommend that psychological distress of HCWs be routinely assessed and that routine counselling, well-being support services, appropriate hazardous leave and insurance be provided to all HCWs.

**Contribution:**

This study adds to the literature on the psychological distress faced by HCWs in South Africa during COVID-19.

## Introduction

December 2019 marked the emergence of the novel coronavirus disease 2019 otherwise known as COVID-19 in Wuhan, China (Zhang et al. 2020). As this infectious disease rapidly spread throughout the world at an alarming rate, it gained global attention and was declared a global pandemic by the World Health Organization (WHO) (WHO 2020; Satici et al. 2021). The first case of COVID-19 in South Africa was reported on 05 March 2020 and subsequently the South African government attempted to mitigate the rate of transmission within the country by adopting prevention measures and precautions to protect human lives (National Department of Health [NdoH] 2020a).

The main preventive measure instituted in South Africa to curb the spread of COVID-19 included a shelter-in-place lockdown. In this instance, all educational institutions and workplaces were closed except for essential services which included emergency services, healthcare, food supply stores and other functions crucial for supporting the economy (Greyling, Rossouw & Adhikari 2021; Department of Cooperative Governance and Traditional Affairs 2020). Work and study from home initiatives were also put in place where possible. In addition, the government mandated the use of face masks by the general public as a compulsory measure as well as the wearing of personal protective equipment (PPE) by healthcare workers (HCWs) and other healthcare professionals (Cook 2020; NDoH 2020b; The Lancet 2020).

Coronavirus disease 2019 has impacted mental health among the general population, with increased rates of psychological distress and mental health disorders being reported (Kola et al. 2021; Kohrt 2021). During the COVID-19 pandemic, high rates of anxiety symptoms, depression and post-traumatic stress disorder were reported in a systematic review (Xiong et al. 2020). Uncertainty and fear about the pandemic as well as the implications of the measures taken to mitigate the spread of COVID-19, which impacted people’s lives and livelihoods and resulted in social isolation, loneliness, confinement, physical inactivity, frustration, boredom, limited access to basic supplies and services, concerns about finances and more, clearly exacerbated the potential increase of mental health disorders as well as an increase in the severity of existing mental health conditions (Moreno et al. 2020; Wettstein et al. 2021). It is in this context that the psychological distress of HCWs should be placed. The COVID-19 pandemic has particularly placed immense pressure on HCWs in the forefront of the burgeoning pandemic, putting their mental health and well-being at risk within an already constrained health system with poor resources (Greenberg et al. 2020; Gupta et al. 2021). Healthcare workers were pressured into finding ways of creating a balance between their physical and mental well-being as well as that of their patients (Greenberg et al. 2020). Healthcare workers are also faced with dual roles which include their roles as healthcare professionals and their responsibility towards their families. These factors therefore play a role on both physical and mental health of HCWs (Greenberg et al. 2020; Koontalay et al. 2021).

During early March 2020, reports from the National Health Commission of China revealed that more than 3300 HCWs were infected with COVID-19 in China, and reports from Italy indicated that 20% of responding HCWs had become infected and some had died (The Lancet 2020). In one district of Gauteng Province, South Africa, at three academic hospitals, incidence of COVID-19 was reported as 2.7 cases per 1000 staff days for nursing staff and 1.1 cases per 1000 staff days for medical doctors (Mdzinwa et al. 2021). Nationally, it was reported that 3.8% COVID-19 admissions in South Africa, from March 2020 to April 2021, were of HCWs (Tlotleng et al. 2022).

It was evident from reports made by HCWs that they were heavily burdened by this pandemic. Healthcare workers shared their experiences of mental and physical exhaustion, experiencing the pain and torment of losing patients and colleagues to the battle of COVID-19, the risk of exposure to infection, and carrying that mental burden that they could also go home and infect their loved ones, which in turn brings about feelings of anxiety (The Lancet 2020). In addition, one also needs to consider the psychological impact of the HCWs as a result of the increased workload (Kisely et al. 2020). In essence, there are numerous factors that play a role when it comes to psychological distress experienced by HCWs on a daily basis including disrupted workflows, increased workload with more time restraints, fear of contracting or passing the virus, being female and occupational protection (Liljestrand & Martin 2021; Muller et al. 2020).

Furthermore, reference can also be made to a similar study that was conducted in Italy that sought out to determine the differences between HCWs and the general population, in terms of behaviour, risk perception and psychological distress related to COVID-19 (Simione & Gnagnarella 2020). Compared to the general population, the study revealed that the HCWs in Italy reported higher risk perception, level of worry and knowledge of COVID-19 infection (Simione & Gnagnarella 2020). In addition, similar results were revealed in other studies that also found that HCWs experienced high levels of anxiety, fear, distress, insomnia and depression (Aly et al. 2021; Shaukat, Ali & Razzak 2020). According to Aly et al. (2021), female HCWs and nurses were more likely to be affected as a result of mental health consequences. This further illustrates HCWs’ vulnerability to psychological distress.

This study presents benchmarked data of HCWs from across South Africa. The objectives of this study were to determine the prevalence of psychological distress as measured by the Kessler (K-10) psychological distress scale among HCWs in South Africa during the COVID-19 pandemic and to determine the factors associated with psychological distress among HCWs at a national level. We also make relevant recommendations to put in place measures for their psychological well-being.

## Methods

### Study approach and design

A cross-sectional study design was used. Participants completed an online survey held on a data-free platform (Manyaapelo et al. 2021; Naidoo et al. 2020).

### Study respondents

The respondents in this study were male and female HCWs in South Africa and aged 18 years and older. The HCWs ranged from several categories, including nurses (all nursing categories), medical practitioners (general practitioners and specialists) and other healthcare professionals (including pharmacists, dental practitioners, optometrists, physiotherapists, dieticians, occupational therapists, radiographers, audio and speech therapists, psychologists, social services practitioners, biokineticists, emergency medical staff, environmental health specialists, medical management staff, orthotists, phlebotomists, podiatrists and research technologists).

### Data collection

Data collection started on 11 April 2020 and continued until 07 May 2020. The survey link was shared widely via social media, email and professional organisations in the health sector. In addition, other media platforms were utilised by the Human Sciences Research Council’s (HSRC’s) research team to encourage participation in the study. The survey was administered online through a data-free Moya Messaging platform, as operated by the HSRC research partner biNu. This mobile telephone and tablet-based application is available on all major application stores free of charge and allows users free access to its content. This end user data-free model allowed anyone with a mobile telephone and tablet-based to participate, regardless of availability of airtime or data credits, thus potentially reaching more respondents. All respondents were encouraged to share the survey link.

Respondents in the survey provided consent via the online platform prior to proceeding to the questionnaire. If consent was not provided, the respondent was thanked for their time and the session was ended. In this case, the questionnaire page did not load. When consent was provided, the questionnaire page loaded and the respondent was presented with 117 closed-ended questions.

### Measures

#### Outcome measure

The main outcome measure of this study, psychological distress, was derived from the 10-item Kessler psychological distress scale (K-10) (Kessler et al. 2002). The scale measures current nonspecific psychological distress and has been validated in the South African context (Andersen et al. 2011). The scale was dichotomised into two categories with a total score < 20 for minimal psychological distress (coded 0) and over 20 for mild to severe psychological distress (coded 1) (Andrews & Slade 2001). Cronbach’s alpha for the psychological distress scale used during this study is α = 0.94, indicating high inter-item reliability.

#### Sociodemographic measures

Sociodemographic variables included sex (male, female), age (18–29 years, 30–39 years, 40–49 years, 50–59 years and ≥ 60 years), population group (black African people, white people, mixed race people and people of Indian or Asian descent), highest level of education (diploma[s] or occupational certificate[s], bachelor’s degree, honours or postgraduate diploma, Master’s degree, specialist qualification and doctorate), public work sector (yes or no), private work sector (yes or no), other work sector (yes or no), province of residence (all nine South African provinces) and geographical type (urban formal, urban informal [informal settlements, peri-urban areas], rural formal [commercial farm areas] and rural informal [tribal authority areas]). It is important to note that mixed race is a racial classification of *South Africa’s Apartheid Government Act 30 of 1950*.

#### Health-related measures in response to COVID-19

Respondents were asked about their perceptions of risk to COVID-19 and reported whether they currently believed their risk to be low, moderate or high. Respondents were also asked whether they think wearing the N95 respirator mask or a surgical mask all the time at work will protect them from contracting the virus (yes, no or don’t know). Questions also included if they were to test positive or have already tested positive for COVID-19, and what their main concerns would be, which included ‘I do not have leave for 21 days (yes or no)’, ‘I have no self-quarantine space at home (yes or no)’, ‘I have no risk pay (yes or no)’ and ‘My life insurance does not cover COVID-19 (yes or no)’. Respondents were also asked if they ‘Have treated or provided care for a patient diagnosed with COVID-19 (yes or no)’, as well as if they ‘Know someone close to you who has been diagnosed with COVID-19 (yes or no)’. Additionally, respondents were asked if there were any well-being support services available to them through their work (yes, no or don’t know), should HCWs get routine counselling during this pandemic (yes, no or don’t know) and whether respondents feel that the South African health system is able to cope with the COVID-19 outbreak (yes, no or don’t know).

### Statistical analysis

Data were benchmarked to the national population of healthcare professionals in South Africa, using estimates from healthcare professional bodies. This process was conducted to increase generalisability of the findings to healthcare professionals across the country. Data were analysed in Stata version 15.0 (StataCorp 2017). Descriptive statistics with unweighted frequencies and weighted percentages were presented. Differences in psychological distress across categories of the independent variables were compared using 95% confidence intervals and chi-square tests. The association between psychological distress and potential explanatory variables was assessed using univariate logistic regression models. All variables found to be significant in the univariate logistic regressions were entered into the multiple logistic regressions. All multiple regression models controlled for age and gender. Crude and adjusted odds ratio (AOR) with 95% confidence intervals and a *P* < 0.05 were considered statistically significant.

### Ethical considerations

Ethics approval was obtained from the Human Sciences Research Council Research Ethics Committee with protocol approval number (REC: 5/03/20). Participation in the survey was voluntary and no personal information was collected from respondents. Participants were informed of their voluntary participation, that their responses were anonymous and that they could easily withdraw from the survey at any given time. Following informed consent on the entry page, participants were automatically directed to the questionnaire. Prior to analysis, all internet protocol (IP) addresses were removed from the data.

## Results

A description of the study sample with weighted percentages is presented in Table 1. A total of 1760 nurses, 2843 medical practitioners and 3004 other healthcare practitioners participated in this study (see Table 1), with approximately 71% of the total sample being female. Most of the nursing and medical practitioner respondents were between 30 and 39 years of age (27% and 31%, respectively). The majority of the sample for nurses (73%), medical practitioners (55%) and other healthcare professionals (57%) consisted of people who identified as black African. Most worked in an urban formal locality (58% – 64%), had high risk perception (49% – 73%), thought that wearing an N95 respirator mask or a surgical mask all the time at work will protect them from contracting COVID-19 (43% – 55%), believed that HCWs should get routine counselling during this pandemic (81% – 94%) and felt that the South African health system is not able to cope with the COVID-19 outbreak (85% – 66%). Most of the nurses (36%), medical practitioners (25%) and other healthcare professionals (27%) stated that their main concern as an HCW, if they have already or should test positive for COVID-19, was that they do not have ‘risk pay’.

**TABLE 1:** Description of the healthcare workers sample, South Africa, 2020.

Variable	Nurses (*N* = 1760)			Medical practitioners (*N* = 2843)			Other healthcare professionals (*N* = 3004)		
	*n*	%†	95% CI	*n*	%†	95% CI	*n*	%†	95% CI
**Gender**									
Female	1566	91.5	90.0–92.9	1481	61.3	58.6–64.0	2309	78.1	75.8–80.2
Male	179	8.4	7.1–10.0	1341	38.6	36.0–41.3	674	21.9	19.8–24.1
**Age (years)**									
18–29	208	13.1	11.1–15.3	382	16.4	14.3–18.8	760	29.8	27.3–32.5
30–39	530	26.9	24.4–29.5	826	30.6	28.0–33.3	973	29.0	26.6–31.4
40–49	493	26.7	24.2–29.4	714	21.5	19.3–23.9	697	21.7	19.5–24.1
50–59	381	23.5	20.8–26.5	474	15.7	13.7–17.9	377	12.2	10.5–14.1
≥ 60	148	9.8	7.8–12.3	447	15.8	13.8–18.1	197	7.3	5.8–9.1
**Population group**									
Black African people	743	73.3	71.1–75.5	495	54.7	51.9–57.4	578	57.2	54.6–59.7
White people	529	10.4	9.4–11.6	1417	26.1	24.3–28.0	1564	25.1	23.4–26.8
Mixed race people	243	13.3	11.6–15.2	193	9.5	8.1–11.0	246	11.2	9.8–12.8
Indian or Asian people	146	2.6	2.2–3.1	387	7.9	7.0–8.8	328	5.6	4.9–6.3
Other people	52	0.3	0.2–0.4	256	1.8	1.6–2.1	161	1.0	0.8–1.2
**Highest level of education**									
Diploma or occupational certificate	758	43.9	40.9–47.0	220	8.1	6.8–9.6	389	17.2	15.2–19.3
Bachelor’s degree	316	19.8	17.3–22.4	903	40.2	37.3–43.1	994	37.2	34.5–40.0
Honours or postgraduate diploma	299	15.3	13.3–17.6	284	10.4	8.8–12.3	719	22.1	19.9–24.5
Master’s degree	149	8.2	6.6–10.1	340	11.0	9.3–12.9	600	17.8	15.8–19.9
Specialist qualification	150	8.9	7.2–10.8	857	26.3	23.9–28.8	50	1.6	1.1–2.4
Doctorate	41	4.0	2.6–6.0	144	4.1	3.1–5.3	125	4.1	3.1–5.3
**Work sector – public‡**									
No	922	44.2	41.2–47.3	1513	44.7	41.9–47.5	2114	62.3	59.4–65.1
Yes	790	55.8	52.7–58.8	1233	55.3	52.5–58.1	759	37.7	34.9–40.6
**Work sector – private‡**									
No	1062	73.1	70.5–75.6	1507	64.6	61.9–67.2	1577	65.0	62.5–67.6
Yes	650	26.9	24.4–29.5	1239	35.4	32.8–38.1	1296	35.0	32.4–37.5
**Work sector – other§**									
No	1320	75.7	72.8–78.5	2034	74.6	72.1–76.9	1770	63.8	61.1–66.4
Yes	392	24.3	21.5–27.2	712	25.4	23.1–27.9	1103	36.2	33.6–38.9
**In which province do you work?**									
Eastern Cape	142	9.6	7.9–11.6	193	10.3	8.6–12.3	201	7.5	6.2–9.0
Free State	60	5.1	3.7–7.0	94	4.9	3.7–6.4	97	5.2	4.0–6.6
Gauteng	413	23.0	20.6–25.6	932	32.4	29.8–35.0	1079	33.2	30.8–35.7
KwaZulu-Natal	484	29.7	27.0–32.6	459	18.2	16.1–20.6	442	15.0	13.2–16.9
Limpopo	40	5.5	3.9–7.8	67	8.7	6.7–11.3	98	12.6	10.2–15.5
Mpumalanga	49	5.4	3.9–7.4	50	3.4	2.3–5.1	99	6.9	5.4–8.8
North West	79	6.6	5.0–8.5	73	5.0	3.8–6.7	92	5.2	4.0–6.7
Northern Cape	19	1.5	0.9–2.5	44	1.8	1.2–2.7	40	1.6	1.1–2.4
Western Cape	427	13.6	12.1–15.3	836	15.3	13.9–16.8	729	12.9	11.7–14.2
**Locality in which you work**									
Urban formal	1147	57.9	54.8–61.0	2029	63.6	60.6–66.4	2170	62.8	59.9–65.7
Urban informal (informal settlements, peri-urban areas)	314	22.8	20.3–25.6	453	23.5	21.0–26.2	403	18.7	16.6–21.0
Rural formal (commercial farm areas)	113	8.1	6.4–10.2	174	7.3	5.9–9.1	156	7.5	6.0–9.4
Rural informal (tribal authority areas)	125	11.2	9.2–13.4	75	5.6	4.2–7.4	124	10.9	8.9–13.5
**Personal risk perception**									
Low	129	7.9	6.2–10.0	264	9.9	8.3–11.8	454	16.0	14.0–18.2
Moderate	307	19.5	16.9–22.4	753	29.1	26.4–31.9	776	34.8	31.8–38.0
High	827	72.6	69.3–75.7	1216	61.0	58.0–64.0	853	49.2	45.9–52.5
**Do you think that wearing an N95 respirator mask or a surgical mask all the time at work will protect you from contracting the virus?**									
Yes	613	54.9	51.3–58.5	859	43.4	40.2–46.7	763	44.3	41.0–47.7
No	470	32.0	28.8–35.4	1068	43.0	39.8–46.2	901	39.8	36.7–43.0
Do not know	163	13.0	10.8–15.7	292	13.6	11.5–16.0	393	15.9	13.7–18.2
**Main concerns as a healthcare worker if you have already or should test positive for COVID-19:**									
I do not have leave for 21 days.	394	18.6	16.5–21.1	584	18.8	16.7–21.1	637	20.7	18.6–23.0
I have no self-quarantine space at home.	513	32.3	29.5–35.3	539	20.5	18.3–23.0	616	25.3	22.8–27.9
I have no risk pay.	597	35.6	32.7–38.6	708	25.1	22.7–27.6	837	27.4	25.1–29.9
My life insurance does not cover COVID-19.	320	18.8	16.5–21.4	331	12.8	11.0–14.9	409	16.6	14.5–18.9
Have treated or provided care for a patient diagnosed with COVID-19.	227	14.0	11.8–16.4	413	13.5	11.7–15.4	162	8.1	6.4–10.1
Know someone close to you who has been diagnosed with COVID-19.	285	19.5	16.8–22.4	618	25.6	22.8–28.5	392	16.2	14.0–18.5
**Are there well-being support services available to you through your work?**									
Yes	581	41.5	37.9–45.2	905	38.4	35.3–41.6	819	40.4	37.2–43.8
No	475	47.0	43.2–50.8	833	42.3	39.0–45.7	849	43.5	40.2–46.9
Do not know	137	11.5	9.2–14.3	405	19.3	16.8–22.1	326	16.1	13.8–18.7
**Should healthcare workers get routine counselling during this pandemic?**									
Yes	1084	93.6	91.3–95.3	1526	80.6	78.3–82.7	1649	88.7	86.9–90.4
No	33	2.5	1.6–4.0	281	8.9	7.5–10.4	105	4.6	3.5–6.1
Do not know	50	3.9	2.6–5.9	313	10.6	9.0–12.3	219	6.7	5.6–8.0
**Do you feel that the South African health system is able to cope with the COVID-19 outbreak?**									
Yes	242	24.7	21.4–28.4	302	15.6	13.3–18.1	384	25.0	22.1–28.1
No	747	60.8	57.0–64.6	1423	66.1	62.8–69.2	1234	58.2	54.8–61.5
Do not know	181	14.4	12.0–17.3	399	18.4	15.9–21.2	362	16.8	14.5–19.5

† , Data were benchmarked to the national population of healthcare professionals in South Africa, using estimates from healthcare professional bodies;

‡ , Weighted percentage;

§ , Categories of work sector were not mutually exclusive. For example, a respondent could work in both the public and private sectors.

Table 2 shows the prevalence of psychological distress among HCWs in South Africa by sociodemographic and health-related variables. Overall, half of the nurses (50.3%), two-fifths of the medical practitioners (40.6%) and just under half of the other healthcare professionals (47.4%) were classified as psychologically distressed. Significant differences for all three categories of HCWs were seen for age, working in the public sector, personal risk perception, not having 21 days of leave, the availability of well-being support services through their place of work, the belief that HCWs get routine counselling during this pandemic and that the South African health system is able to cope with the COVID-19 outbreak.

**TABLE 2:** Prevalence of psychological distress of healthcare workers by sociodemographic and COVID-19 variables, South Africa, 2020.

Variable	Nurses			Medical practitioners			Other healthcare professionals		
	%	95% CI	*P*	%	95% CI	*P*	%	95% CI	*P*
Total	50.3	46.5–54.1	-	40.6	37.4–43.9	-	47.4	44.0–50.8	-
**Gender**	-	-	0.365	-	-	< 0.001*	-	-	< 0.001*
Female	50.8	46.7–54.8	-	47.2	42.6–51.8	-	50.7	46.8–54.6	-
Male	45.6	35.6–56.0	-	30.3	26.3–34.6	-	36.4	30.3–43.0	-
**Age (years)**	-	-	< 0.001*	-	-	< 0.001*	-	-	< 0.001*
18–29	71.1	59.9–80.3	-	52.0	42.6–61.3	-	57.9	51.4–64.2	-
30–39	61.0	54.4–67.1	-	51.9	45.8–58.0	-	50.1	44.3–55.9	-
40–49	53.3	46.7–59.8	-	40.1	33.7–46.7	-	45.0	37.8–52.3	-
50–59	36.9	29.1–45.5	-	32.9	26.0–40.6	-	33.8	26.1–42.5	-
≥ 60	27.2	16.9–40.6	-	19.3	13.1–27.5	-	24.2	15.1–36.4	-
**Population group**	-	-	0.001*	-	-	0.072	-	-	0.300
Black African people	53.1	48.0–58.2	-	41.4	35.5–47.5	-	46.4	40.6–52.3	-
White people	46.9	41.6–52.3	-	36.3	33.3–39.3	-	46.5	43.3–49.7	-
Mixed race people	37.7	30.3–45.7	-	46.6	38.3–55.1	-	49.5	41.4–57.7	-
Indian or Asian people	58.2	47.8–67.8	-	45.8	39.8–51.9	-	56.6	49.7–63.2	-
Other people	64.5	44.7–80.4	-	32.4	25.7–40.0	-	39.9	29.4–51.6	-
**Highest level of education**	-	-	0.002*	-	-	0.009*	-	-	0.287
Diploma or occupational certificate	53.1	47.5–58.7	-	46.9	36.9–57.1	-	48.2	40.4–56.0	-
Bachelor’s degree	44.3	35.7–53.2	-	46.9	41.3–52.5	-	49.8	43.9–55.7	-
Honours or postgraduate diploma	52.8	44.0–61.5	-	34.4	25.1–45.2	-	46.8	40.2–53.5	-
Master’s degree	37.9	26.3–51.0	-	30.8	23.2–39.6	-	46.5	38.8–54.4	-
Specialist qualification	69.3	57.4–79.0	-	37.1	31.5–43.0	-	43.9	21.9–68.5	-
Doctorate	27.0	11.6–51.1	-	31.4	18.0–48.9	-	28.1	17.3–42.2	-
**Work sector – public**	-	-	< 0.001*	-	-	< 0.001*	-	-	0.020*
No	42.2	37.0–47.6	-	32.4	28.6–36.5	-	43.9	40.1–47.8	-
Yes	56.7	51.4–61.8	-	47.2	42.4–52.1	-	52.5	46.4–58.5	-
**Work sector – private**	-	-	0.744	-	-	< 0.001*	-	-	0.031*
No	50.5	45.9–55.2	-	45.5	41.1–49.8	-	49.9	45.5–54.4	-
Yes	49.2	43.0–55.5	-	31.9	27.7–36.5	-	42.6	37.9–47.5	-
**Work sector – other**	-	-	< 0.001*	-	-	0.918	-	-	0.081
No	54.5	50.2–58.7	-	40.7	36.9–44.5	-	49.5	45.2–53.8	-
Yes	37.8	30.2–46.0	-	40.3	34.3–46.5	-	43.3	38.0–48.8	-
**Province in which you work**	-	-	0.076	-	-	0.692	-	-	0.164
Eastern Cape	47.0	35.1–59.2	-	38.6	28.7–49.5	-	62.1	51.3–71.8	-
Free State	47.5	28.9–66.7	-	37.8	23.1–55.1	-	59.4	44.4–72.7	-
Gauteng	49.4	42.0–56.8	-	42.2	37.1–47.5	-	44.0	39.1–49.1	-
KwaZulu-Natal	58.7	51.9–65.2	-	44.6	36.9–52.7	-	49.0	41.2–56.8	-
Limpopo	46.2	26.2–67.5	-	30.7	17.5–48.2	-	44.5	29.8–60.1	-
Mpumalanga	66.1	44.9–82.3	-	44.0	23.2–67.1	-	43.3	29.9–57.8	-
North West	45.4	30.5–61.2	-	33.7	19.0–52.5	-	54.0	37.9–69.3	-
Northern Cape	36.6	13.5–68.0	-	28.8	13.9–50.3	-	33.7	17.9–54.2	-
Western Cape	37.3	31.0–44.1	-	42.0	37.3–46.9	-	44.7	39.2–50.3	-
**Locality of work**	-	-	0.222	-	-	0.07	-	-	0.122
Urban formal	46.9	42.2–51.6	-	38.3	34.7–42.1	-	44.2	40.5–48.0	-
Urban informal (informal settlements, peri-urban areas)	53.0	44.9–60.9	-	48.9	41.3–56.7	-	55.3	47.5–62.9	-
Rural formal (commercial farm areas)	60.2	46.1–72.8	-	40.8	28.4–54.6	-	56.0	42.4–68.7	-
Rural informal (tribal authority areas)	55.0	41.3–67.9	-	31.7	18.5–48.7	-	47.8	33.8–62.1	-
**Personal risk perception**	-	-	< 0.001*	-	-	0.001*			< 0.001*
Low	25.7	16.2–38.3	-	25.9	18.1–35.6	-	31.5	25.9–37.8	-
Moderate	40.6	33.1–48.5	-	36.7	31.6–42.1	-	45	39.5–50.6	-
High	55.5	50.9–60.0	-	44.7	40.3–49.2	-	54.5	49.3–59.6	-
**Do you think that wearing an N95 respirator mask or a surgical mask all the time at work will protect you from contracting the virus?**	-	-	0.021*	-	-	0.154	-	-	0.754
Yes	48.2	42.9–53.5	-	42.7	37.6–48.0	-	47.3	41.9–52.8	-
No	50.2	44.0–56.3	-	37.1	32.7–41.8	-	46.8	41.8–51.8	-
Do not know	64.2	54.1–73.1	-	45.6	36.7–54.8	-	50.5	42.6–58.2	-
**Main concerns as a healthcare worker if you have already or should test positive for COVID-19. I do not have leave for 21 days.**	-	-	0.031*	-	-	0.001*	-	-	< 0.001*
No	47.9	43.4–52.4	-	37.5	33.8–41.3	-	41.7	37.7–45.7	-
Yes	57	50.0–63.7	-	50.0	43.5–56.5	-	60.8	54.8–66.5	-
**I have no self-quarantine space at home.**	-	-	0.794	-	-	0.092	-	-	0.117
No	49.8	44.8–54.9	-	38.8	35.2–42.6	-	45.3	41.3–49.3	-
Yes	50.9	45.1–56.6	-	45.3	38.8–52.1	-	51.1	45.1–57.1	-
**I have no risk pay.**	-	-	0.046*	-	-	0.419	-	-	0.004*
No	46.4	41.2–51.7	-	39.6	35.7–43.7	-	43.3	39.0–47.8	-
Yes	54.1	48.7–59.5	-	42.5	37.0–48.2	-	53.4	48.2–58.5	-
**My life insurance does not cover COVID-19.**	-	-	0.192	-	-	0.003*	-	-	0.001*
No	48.8	44.4–53.2	-	38.3	34.8–41.9	-	44.1	40.4–47.8	-
Yes	54.6	47.0–62.0	-	52.1	43.8–60.3	-	57.9	50.4–65.0	-
**Have you treated or provided care for a patient diagnosed with COVID-19?**	-	-	0.099	-	-	0.661	-	-	0.147
Yes	57.5	48.7–65.8	-	39.2	32.4–46.4	-	56.2	44.0–67.6	-
No	49.3	45.1–53.5	-	40.9	37.4–44.6	-	46.9	43.4–50.4	-
**Do you know someone close to you who has been diagnosed with COVID-19?**	-	-	0.167	-	-	0.794	-	-	0.011*
Yes	55.7	47.2–63.8	-	41.4	35.2–47.9	-	56.7	49.1–64.0	-
No	49.0	44.8–53.3	-	40.4	36.7–44.3	-	45.8	42.1–49.5	-
**Are there well-being support services available to you through your work?**	-	-	< 0.001*	-	-	< 0.001*	-	-	< 0.001*
Yes	38.3	33.0–43.8	-	32.2	27.8–36.9	-	37.0	32.2–42.0	-
No	62.9	57.3–68.2	-	47.7	42.4–53.1	-	58.1	53.0–63.0	-
Do not know	43.3	32.4–54.8	-	41.7	34.3–49.5	-	45.3	37.3–53.6	-
**Should healthcare workers get routine counselling during this pandemic?**	-	-	< 0.001*	-	-	< 0.001*	-	-	< 0.001*
Yes	52.5	48.5–56.5	-	43.7	39.9–47.6	-	49.3	45.6–53.0	-
No	35.4	17.6–58.4	-	17.5	12.1–24.6	-	24.2	15.5–35.7	-
Do not know	15.9	7.5–30.8	-	35.0	27.8–42.9	-	44.3	35.6–53.4	-
**Do you feel that the South African health system is able to cope with the COVID-19 outbreak?**	-	-	< 0.001*	-	-	0.039*	-	-	< 0.001*
Yes	34.9	27.5–43.0	-	31.8	24.2–40.5	-	35.2	28.7–42.4	-
No	59.0	54.3–63.6	-	43.4	39.4–47.5	-	53.1	48.7–57.4	-
Do not know	42.6	33.3–52.4	-	37.3	30.0–45.2	-	46.4	38.4–54.5	-

* , Significant *P* < 0.05.

For nurses, significant differences were also observed for population group, education level, other work sectors, thinking that wearing an N95 respirator mask or a surgical mask all the time at work will protect them from contracting the virus, and having no risk pay as a concern. For medical practitioners, significant differences were also observed for gender, education level, working in the private sector, and the concern that their life insurance does not cover COVID-19. In terms of other healthcare professionals, significant differences were also observed for gender, working in the private sector, the concern that their life insurance does not cover COVID-19 and having no risk pay, and knowing someone close to them who has been diagnosed with COVID-19.

Table 3 presents the results of the multiple logistic regression models for psychological distress for nurses, medical practitioners and other healthcare professionals. Among nurses, older age played a significant role in determining psychological distress where those who were 40–49 years, 50–59 years and 60 and more years old were significantly less likely to have psychological distress compared to those aged 18–29 years. Nurses with a specialist qualification were two and a half times (AOR 2.53, 95% CI 1.35–4.77, *P* = 0.004) more likely to be distressed when compared to those nurses with diplomas and/or occupational certificates. Nurses who had high personal risk perception were almost two and a half times (AOR 2.47, 95% CI 1.24–4.91, *P* = 0.010) more likely to be distressed when compared to those nurses who had low personal risk perception. Those nurses who stated that there were well-being support services available to them through their work or were unsure of whether those services were available at their workplace were significantly less likely to be distressed. Nurses who thought that the South African health system is able to cope with the COVID-19 outbreak were significantly less likely to be distressed.

**TABLE 3:** Univariate and multiple regression model for psychological distress showing significant variables, South Africa, 2020.

Variable	Multiple regression								
	Nurses			Medical practitioners			Other healthcare practitioners		
	AOR	95%CI	*P*	AOR	95%CI	*P*	AOR	95%CI	*P*
**Gender**									
Male	-	Ref	-	-	Ref	-	-	Ref	-
Female	1.58	0.93–2.68	0.088	1.51	1.1–2.08	0.011*	1.75	1.2–2.55	0.004*
**Age (years)**									
18–29	-	Ref	-	-	Ref	-	-	Ref	-
30–39	0.65	0.35–1.23	0.186	1.08	0.66–1.77	0.750	0.75	0.52–1.08	0.124
40–49	0.51	0.27–0.96	0.037*	0.75	0.43–1.3	0.302	0.6	0.39–0.92	0.019*
50–59	0.32	0.15–0.66	0.002*	0.62	0.34–1.13	0.116	0.42	0.25–0.71	0.001*
≥ 60	0.22	0.09–0.53	0.001*	0.33	0.15–0.7	0.004*	0.23	0.12–0.45	< 0.001*
**Population group**									
Black African people	-	Ref	-	-	-	-	-	Ref	-
White people	1.3	0.8–2.13	0.291	-	-	-	1.57	1.07–2.28	0.020*
Mixed race people	0.69	0.44–1.09	0.110	-	-	-	1.03	0.62–1.7	0.910
Indian or Asian people	1.15	0.69–1.91	0.587	-	-	-	1.4	0.89–2.19	0.147
Other people	2.85	0.91–8.89	0.071	-	-	-	1.08	0.56–2.09	0.826
**Highest level of education**									
Diploma or occupational certificate	-	Ref	-	-	Ref	-	-	Ref	-
Bachelor’s degree	0.85	0.53–1.36	0.492	0.67	0.39–1.16	0.155	0.93	0.58–1.49	0.768
Honours or postgraduate diploma	1.41	0.86–2.31	0.171	0.5	0.24–1.02	0.057	0.74	0.45–1.23	0.246
Master’s degree	1.05	0.57–1.95	0.868	0.58	0.29–1.12	0.106	0.97	0.58–1.6	0.891
Specialist qualification	2.53	1.35–4.77	0.004*	0.79	0.45–1.39	0.415	1.64	0.51–5.3	0.409
Doctorate	1.19	0.36–3.92	0.778	0.77	0.32–1.86	0.559	0.61	0.23–1.6	0.315
**Work sector – public**	1.05	0.72–1.53	0.797	1.41	0.97–2.06	0.073	1.18	0.8–1.74	0.401
**Work sector – private**		-	-	0.86	0.6–1.25	0.439	0.69	0.49–0.98	0.040
**Work sector – other**	0.94	0.61–1.45	0.775	-	-	-	-	-	-
**Province in which you work**									
Eastern Cape	-	-	-	-	-	-	-	Ref	-
Free State	-	-	-	-	-	-	0.99	0.45–2.18	0.981
Gauteng	-	-	-	-	-	-	0.71	0.41–1.23	0.225
KwaZulu-Natal	-	-	-	-	-	-	0.73	0.4–1.33	0.300
Limpopo	-	-	-	-	-	-	0.66	0.29–1.48	0.311
Mpumalanga	-	-	-	-	-	-	0.45	0.21–0.94	0.034*
North West	-	-	-	-	-	-	0.99	0.39–2.52	0.981
Northern Cape	-	-	-	-	-	-	0.3	0.11–0.79	0.015*
Western Cape	-	-	-	-	-	-	0.78	0.45–1.35	0.367
**Locality of work**									
Urban formal	-	-	-	-	Ref	-	-	Ref	-
Urban informal (informal settlements, peri-urban areas)	-	-	-	0.98	0.64–1.49	0.918	1.32	0.86–2	0.201
Rural formal (commercial farm areas)	-	-	-	0.83	0.46–1.53	0.555	1.27	0.69–2.34	0.446
Rural informal (tribal authority areas)	-	-	-	0.43	0.21–0.91	0.027*	0.91	0.45–1.85	0.794
**Personal risk perception**									
Low	-	Ref	-	-	Ref	-	-	Ref	-
Moderate	1.77	0.88–3.54	0.108	1.33	0.76–2.32	0.311	1.43	0.96–2.14	0.080
High	2.47	1.24–4.91	0.010*	1.65	0.96–2.83	0.069	2.09	1.4–3.12	< 0.001*
**Do you think that wearing an N95 respirator mask or surgical mask all the tim e at work will protect you from contracting the virus?**									
No	-	Ref	-	-	-	-	-	-	-
Yes	0.87	0.6–1.27	0.461	-	-	-	-	-	-
Do not know	1.6	0.93–2.76	0.089	-	-	-	-	-	-
**Main concerns as a healthcare worker if you have already or should test positive for COVID-19:**									
I do not have leave for 21 days	1.21	0.83–1.76	0.320	1.51	1.08–2.09	0.015*	1.71	1.26–2.33	0.001*
I have no self-quarantine space at home	-	-	-		-	-	-	-	-
I have no risk pay	0.94	0.67–1.33	0.735		-	-	1.23	0.91–1.67	0.173
My life insurance does not cover COVID-19	-	-	-	1.57	1.05–2.32	0.026*	1.78	1.25–2.53	0.001*
Treated or provided care for a patient diagnosed with COVID-19	-	-	-		-	-	-	-	-
Someone close to you who has been diagnosed with COVID-19	-	-	-		-	-	1.55	1.1–2.19	0.013*
**Are there well-being support services available to you through your work?**									
No	-	Ref	-		Ref	-	-	Ref	-
Yes	0.53	0.36–0.78	0.001*	0.5	0.36–0.69	< 0.001*	0.44	0.32–0.61	< 0.001*
Do not know	0.55	0.32–0.95	0.031*	0.66	0.42–1.04	0.074	0.53	0.35–0.79	0.002*
**Should healthcare workers get routine counselling during this pandemic?**									
No	-	-	-	-	Ref	-	-	Ref	-
Yes	-	-	-	2.51	1.6–3.93	< 0.001*	3.82	1.85–7.89	< 0.001*
Do not know	-	-	-	2.18	1.27–3.76	0.005*	3.14	1.36–7.23	0.007*
**Do you feel that the South African health system is able to cope with the COVID-19 outbreak?**									
No	-	Ref	-	-	Ref	-	-	Ref	-
Yes	0.65	0.42–0.99	0.047*	0.75	0.46–1.2	0.222	0.6	0.41–0.88	0.010*
Do not know	0.64	0.4–1.01	0.054	0.93	0.64–1.36	0.719	0.99	0.67–1.46	0.955

AOR, adjusted odds ratio.

* , Significant *P* < 0.05.

Among medical practitioners, females were one and a half times (AOR 1.51, 95% CI 1.10–2.08, *P* = 0.011) more likely to be psychologically distressed than their male counterparts, while those who aged 60 years or older were significantly less likely to have distressed than 18–29-year olds. Medical practitioners who worked in rural informal areas were significantly less likely to have psychological distress than those who worked in urban formal areas (AOR 0.43, 95% CI 0.21–0.91, *P* = 0.027). Medical practitioners who had a concern regarding not having 21 days of leave available and regarding their life insurance not covering COVID-19 were significantly more likely to be distressed. Those who stated that there are well-being support services available to them through their work were significantly less likely to be distressed. Those who stated that HCWs should get routine counselling during this pandemic were two and a half times (AOR 2.51, 95% CI 1.60–3.93, *P* < 0.001) more likely to be distressed and those who were unsure about whether they should get counselling were also significantly more likely to be distressed (AOR 2.18, 95% CI 1.27–3.76, *P* = 0.005).

Among other healthcare professionals, females were 1.75 times (AOR 1.75, 95% CI 1.2–2.55, *P* = 0.004) more likely to be psychologically distressed than their male counterparts and those who were 40–49, 50–59 and 60 years or older were significantly less likely to suffer distress compared to those aged 18–29 years. Psychological distress was significantly higher among other healthcare professionals from the white population group than those from the black African population group. We noted provincial differences as well, where other healthcare professionals working in Mpumalanga and Northern Cape were significantly less likely to be distressed than those in the Eastern Cape. Other healthcare professionals who had high personal risk perception were more than twice (AOR 2.09, 95% CI 1.40–3.12, *P* < 0.001) as likely to be distressed when compared to their colleagues who had low personal risk perception. Those who had a concern of not having 21 days of leave available, those whose life insurance did not cover COVID-19 and those who had someone close to them who had been diagnosed with COVID-19 were significantly more likely to be distressed. Other healthcare professionals who reported that there were well-being support services available to them through their work or that they did not know whether these services were available to them were significantly less likely to have psychological distress. Other healthcare professionals who stated that HCWs should get routine counselling during this pandemic and those who were unsure whether they should get routine counselling were almost four times (AOR 3.82, 95% CI 1.85–7.89, *P* < 0.001 and AOR 3.14, 95% CI 1.36–7.23, *P* = 0.007, respectively) more likely to be distressed, and those who felt that the South African health system is able to cope with the COVID-19 outbreak were significantly less likely to be distressed.

## Discussion

The study sought to utilise national benchmarked HCW data to ascertain the prevalence of psychological distress among nurses, medical practitioners and other healthcare professionals in South Africa during the COVID-19 pandemic as well as to determine the factors associated with psychological distress among these three categories of HCWs at a national level. This study found that half of the nurses, two-fifths of the medical practitioners and just under half of the other healthcare professionals were classified as psychologically distressed according to the 10-item psychological distress scale (Kessler et al. 2002). This finding is of great concern, as psychological distress among HCWs in South Africa during the COVID-19 pandemic seems exceptionally high. As there are no other psychological distress studies among South African HCWs to compare this finding to, we compared to a 2012 South African general population survey that found psychological distress at 24% (Mthembu et al. 2017). The prevalence findings of this study could point to an increased burden carried by HCWs. It must be noted though that this HCW study was conducted during April and May 2020, when the COVID-19 outbreak was relatively new, where community transmission was rife and the number of daily cases was rising steeply. During this time, there was a heightened awareness and panic in the country and in the healthcare system and this could explain the heightened psychological distress found in this study.

Globally, HCWs across both private and public sectors have faced the realities of being at the forefront of the COVID-19 pandemic with reports of the mental health toll on HCWs being reported during this global health crisis (Huang et al. 2020; Liu et al. 2020; Tsamakis et al. 2020). Studies conducted among HCWs have indicated that they have experienced poor mental health both during and post epidemics, including post-traumatic stress, burnout, depression and anxiety (Lancee, Maunder & Goldbloom 2008; Maunder et al. 2006; Park et al. 2018). A study conducted during the severe acute respiratory syndrome (SARS) epidemic found that more than 75% of HCWs experienced some kind of psychiatric morbidity (Philip & Cherian 2020).

In South Africa, this study found several determinants of psychological distress among HCWs. We found that female medical practitioners and females in other healthcare professions were significantly more likely to be psychologically distressed than their male counterparts. These findings echo global findings among HCWs during pandemic periods. Two studies that were conducted during the SARS epidemic (Chong et al. 2004) as well as two studies conducted during the COVID-19 pandemic found that females experienced greater psychological distress than that of their male counterparts (Lai et al. 2020). In addition, being female was a major risk factor for increased risk of mental health problems (Davico et al. 2020; Huang et al. 2020; Lai et al. 2020; Zhang et al. 2020); in fact, it is noted that females have higher psychological distress than males in general South African population studies (Mthembu et al. 2017).

Across all HCWs that took part in the study, age was a determinant of psychological distress, with younger HCWs, between the ages of 18 and 29 years, being more likely to experience psychological distress compared to older HCWs. This is consistent with findings from China following the COVID-19 and SARS pandemics, where a younger age was associated with greater ‘depressive symptomatology’, while older HCWs experienced less psychological distress compared with younger HCWs (Liu et al. 2012, 2020).

Nurses and other healthcare professionals with high risk perceptions of becoming infected with COVID-19 were more than twice as likely to be distressed when compared to their colleagues who had low personal risk perception. The study did not assess the actual level of exposure to COVID-19 risk among HCWs; however, risk perceptions may be reflective of risk exposure as well as heightened awareness, panic and perceived loss of control (Abid et al. 2020). Risk factors specific to the unique occupational activities faced by the healthcare workers as well as the organisational support they believed they either had or did not have played a role in the level of psychological distress. The concern regarding not having 21 days of leave available and regarding life insurance not covering COVID-19 condition contributed significantly to the psychological distress of medical practitioners and other healthcare professionals. These concerns were warranted given the infectious nature of COVID-19 and given that new information regarding this disease was rapidly evolving during the course of the study. As COVID-19 was novel, it was not known if life and/or death insurance would be paid if an HCW contracted COVID-19 under hazardous working conditions. The concern was that those in the frontline were not only placing their life at risk but also placing their family’s potential future income and/or livelihoods at risk should the medical practitioners and other healthcare professionals die due to COVID-19.

Other healthcare professionals who had someone close to them diagnosed with COVID-19 were significantly more likely to be distressed and other healthcare professionals who stated that HCWs should get routine counselling during this pandemic and those who were unsure whether they should get routine counselling were almost four times more likely to be distressed. This distress could raise the issue of HCWs experiencing ‘moral injury’ where psychological distress results from actions or lack thereof which violate one’s moral or ethical code (Williamson et al. 2020). The difficult situations that HCWs find themselves in where their best efforts are not enough for their patients and colleagues are the seeds of a moral injury. This is relevant to the experiences of healthcare professionals across the world given the unparalleled situations they find themselves in with respect to provision of care and treatment during a global pandemic. In such situations, mental health services are crucial to support HCWs’ psychological health. In a study undertaken in New York City among HCWs, it was reported that among nurses and advanced practice providers, they expressed interest in additional wellness resources to mitigate stress (Shechter et al. 2020). This is notable as all levels of HCWs in this study who reported that there were well-being support services available to them through their work were significantly less likely to have psychological distress.

A few determinants of increased psychological distress were noted in this survey and require further investigation to understand and explain their complexities. These include higher psychological distress among the white population in the other healthcare professionals category and among nurses with a specialist qualification. This finding is consistent with findings in New York among nurses and advanced practice providers, who were significantly more likely to be screened positive for acute stress and symptoms of depression (Shechter et al. 2020). Among other healthcare professionals, those working in Mpumalanga and Northern Cape provinces were significantly less likely to be distressed than those in the Eastern Cape Province, and medical practitioners in rural informal areas were significantly less likely to have psychological distress than those who worked in urban formal areas. Interestingly, those who felt that or did not know if the South African health system is able to cope with the COVID-19 outbreak were also significantly less likely to be distressed.

## Limitations

It is important to emphasise that the methodology of this study relied on HCWs to self-complete the questionnaire on an online platform and thus biased the sample as only those that wanted to and had the time to complete the survey did so. This survey only utilised one measure for mental health, namely the 10-item Kessler psychological distress scale (Kessler et al. 2002). The cross-sectional nature of the study limits causational interpretations.

## Conclusion and recommendations

The psychological state of frontline workers is at risk. The COVID-19 pandemic has both burdened healthcare systems and had adverse psychological impact on the HCWs who serve on the frontline (Muller et al. 2020). South Africa reflects the global situation with the majority of HCWs experiencing high levels of psychological distress. The WHO has placed an emphasis on the excessive burden placed on frontline workers during the COVID-19 pandemic and has called for action to address and implement measures to address the urgent needs to save lives and prevent a serious impact on both the physical and mental health of HCWs (WHO 2020).

In order to successfully face this global health crisis for a prolonged period of time, frontline workers need to be protected to ensure sustainability of the workforce (Godlee 2020; Remuzzi & Remuzzi 2020). However, the findings globally as well as in South Africa indicate that psychological distress among HCWs demonstrates that the healthcare system is not able to protect those on the frontline. Understanding these unique risks as well as the mental health impact(s) that HCWs encounter on a daily basis is important so that we can identify potential interventions to address these effects (Muller et al. 2020).

We recommend that the psychological state of all HCWs in South Africa be routinely assessed. Routine counselling and well-being support services should be provided to all HCWs in South Africa, especially female HCWs, irrespective of the global pandemic. Healthcare workers should be given assurances that their health would be prioritised, without a financial cost to them, should they fall ill due to hazardous working conditions, and that their families would also be protected financially should something happen to them due to the nature of their work. As the study found that older HCWs were less likely to be distressed than their younger counterparts, it would be important to engage with these older HCWs and utilise them as mentors to younger HCWs to aid them in their psychological distress. As much as the HCWs support a country, we in turn need to support our HCWs.
